# Editorial: Diagnosis, Treatment, and Prognosis of Viral Hepatitis

**DOI:** 10.3389/fmed.2022.882878

**Published:** 2022-04-28

**Authors:** Jian Wu, Yijin Wang, Chuanlong Zhu, Wenyu Lin

**Affiliations:** ^1^Department of Clinical Laboratory, The Affiliated Suzhou Hospital of Nanjing Medical University, Suzhou Municipal Hospital, Gusu School, Nanjing Medical University, Jiangsu, China; ^2^State Key Laboratory for the Diagnosis and Treatment of Infectious Diseases, National Clinical Research Center for Infectious Diseases, The First Affiliated Hospital, Zhejiang University School of Medicine, Hangzhou, China; ^3^School of Medicine, Southern University of Science and Technology, Shenzhen, China; ^4^Department of Infectious Disease, The First Affiliated Hospital of Nanjing Medical University, Nanjing, China; ^5^Liver Center, Massachusetts General Hospital, Harvard Medical School, Boston, MA, United States

**Keywords:** viral hepatitis, diagnosis, prognosis, treatment, host immune response

Viral hepatitis is an infectious disease mainly manifested by liver lesions caused by various hepatitis viruses, including hepatitis A virus (HAV), hepatitis B virus (HBV), hepatitis C virus (HCV), hepatitis D virus (HDV), and hepatitis E virus (HEV). Viral hepatitis poses a huge threat to human public health (1). In 2010, the 63rd World Health Assembly (WHA) adopted a resolution (WHA63.18) (2) which stated that ~2 billion people worldwide are infected with HBV, and about 350 million people are chronically infected with hepatitis B. Nowadays, hepatitis C is still not preventable by vaccines, and almost 80% of HCV infections are chronic infections. Given that viral hepatitis is a serious global public health problem, we call on governments, Parties and the population to scale up efforts to prevent, diagnose, and treat viral hepatitis in the control of viral hepatitis.

In recent years, the world has made impressive progress in a number of areas, including innovations in hepatitis treatment and the expansion of HBV vaccine immunization to prevent new HBV infections. However, until 2021, there are still 296 million people with chronic HBV infection, 58 million people with HCV infection. 1.1 million people die from HBV and HCV infection every year (2), and 3 million people are still newly infected with HBV and HCV. Only 30.4 million (10%) patients with chronic hepatitis B were diagnosed, of whom 6.6 million (22%) received antiviral therapy. 15.2 million (21%) people with hepatitis C infection were diagnosed, of which 9.4 million (62%) received antiretroviral therapy (3). The diagnosis of viral hepatitis patients, the evaluation of patient prognosis and the exploration of novel markers of antiviral efficacy are urgent problems to be solved. For a long time in the future, this field would be advanced by leaps and bounds (4).

Therefore, the appearance of this special issue is very timely. This Research Topic aims to collect articles or reviews that provide insights into disease understanding and translational potential for better clinical care. Early diagnosis of hepatitis infection and early assessment of its prognosis are critical for effective treatment and care (5, 6). In this special issue, Zhang et al. evaluated the diagnostic value of adenosine deaminase, α-l-fucosidase, and lactic acid in liver cirrhosis and hepatocellular carcinoma related to hepatitis B. Through the cohort analysis, Ding et al. constructed a novel non-invasive model for the predication of liver fibrosis with chronic hepatitis B. Genetic mutations in TNFSF11 were proved to be associated with the chronicity of hepatitis C by Huang et al.. Guo et al. identified three genes through bioinformatics, and used them to establish immune-related prognostic characteristics for patients with HCV-related cirrhosis. The correlation between protein induced by vitamin K absence or antagonist-II and trend of changes in hepatitis E patients was also revealed by Chen et al. Nowadays, the application of exosome and multi-omics technology have strongly promoted the primary screening and early intervention of viral hepatitis. Sun et al. devoted into bile acid metabolism associated with HBV infection through the transcriptome and gut microbiome. Cui et al. reviewed the advances in muti-omics applications in hepatocellular carcinoma. In addition, Zhou H. et al. summarized the role of exosomes in viral hepatitis. Tong et al. also investigated the prognostic value of serum exosomal AHCY expression in liver cirrhosis associated with hepatitis B.

Host manifestations in hepatitis viruses have always been a fundamental issue and focus of controversy in this research field, involving the severe mechanism of viral hepatitis and antiviral efficacy (7, 8). In this special issue, Gong et al. discussed the role of Th22 cells in human viral diseases. Also, Jin et al. focused on the peripheral immune cell exhaustion in patients with chronic hepatitis B. Finally, the therapeutic effect of viral hepatitis has also been evaluated. Cheng et al. concluded the novel treatment choices for HBV and human immunodeficiency virus (HIV) co-infection patients. Zhou J. et al. also summarized the antiviral therapy for chronic HBV infection with persistently normal alanine aminotransferase. Additionally, sirtuins were also considered as the potential therapeutic targets for HBV infection by Kong et al., all of which may provide new insight into treatment choices in viral hepatitis.

This Research Topic aims to collect articles or reviews that provide insights into disease understanding and translational potential for better clinical care. Finally, we hope you enjoy reading this special issue.

## Author Contributions

All authors listed have made a substantial, direct, and intellectual contribution to the work and approved it for publication.

## Conflict of Interest

The authors declare that the research was conducted in the absence of any commercial or financial relationships that could be construed as a potential conflict of interest.

## Publisher's Note

All claims expressed in this article are solely those of the authors and do not necessarily represent those of their affiliated organizations, or those of the publisher, the editors and the reviewers. Any product that may be evaluated in this article, or claim that may be made by its manufacturer, is not guaranteed or endorsed by the publisher.
